# Whether Weather Matters with Migraine

**DOI:** 10.1007/s11916-024-01216-8

**Published:** 2024-02-15

**Authors:** Delora Elizabeth Denney, Jane Lee, Shivang Joshi

**Affiliations:** 1 University of Mississippi Medical Center, 2500 N State Street, Jackson, MS 39213 USA; 2 North Shore University Hospital/Long Island Jewish Hospital, 300 Community Drive, Manhasset, NY 11030 USA; 3 Community Neuroscience Services, Westborough, MA USA

**Keywords:** Weather, Migraine, Headache, Pain

## Abstract

**Purpose of Review:**

Many patients with migraine report their attacks are triggered by various weather anomalies. Studies have shown mixed results regarding the association of migraine to weather changes. The purpose of the current review is to compile the most up-to-date research studies on how weather may affect migraine. In addition, we explore the association between weather and other inflammatory disease states as well as neurotransmitters.

**Recent Findings:**

Migraine attacks can be related to weather variables such as barometric pressure, humidity, and wind. However, the results of recent studies are inconsistent; weathers’ effect on migraine attacks is around 20%. However, very strong weather factors have a more significant effect on migraine attack variables.

**Summary:**

Many individuals identify weather as a migraine attack trigger, yet we see no causative relationship between weather and migraine patterns. The outcomes of studies indicate mixed results and reflect individual variation in how weather can impact migraine patterns. Similar relationships can be seen with other rheumatologic and pain conditions in general. Overall, the combination of weather plus other factors appears to be a more significant migraine trigger.

## Introduction

Migraine is one of most prevalent neurological disorders and associated with significant global disability. According to the global burden of disease study in 2019 (GBD 2019), migraine ranked as the second most common cause of disability in all genders and ages. It is ranked as the first cause of world’s disability in young women [1]. In most of the literature, those with migraine describe various triggers as either endogenous events or exogenous stimuli that increase the probability of a migraine attack. In a meta-analysis performed by Pellegrino et al., 27,122 participants rated weather as one of the top 4 triggers for their primary headache [2].

There are many components of weather that are reported to affect migraine such as barometric pressure, humidity, temperature, and seasons. In the International Classification of Headache Disorders (ICDH-3), there is a primary headache diagnosis attributed to change in a weather component. The high-altitude headache is described as “bilateral and aggravated by exertion with ascent above 2,500 m.” This resolves within 24 h after ascent [3]. However, there is minimal information available for other changes in weather conditions. There is inconsistent reporting in literature regarding directionality of the relationship between weather and migraine. This article aims to discuss up-to-date research that delineates the relationship between migraine attack occurrences and weather changes. In addition, we will review literature describing different weather conditions that may impact neurotransmitter changes, resulting in clinical symptoms of migraine. This may be an important relationship to further explore.

## Barometric Pressure

**Fig. 1:**
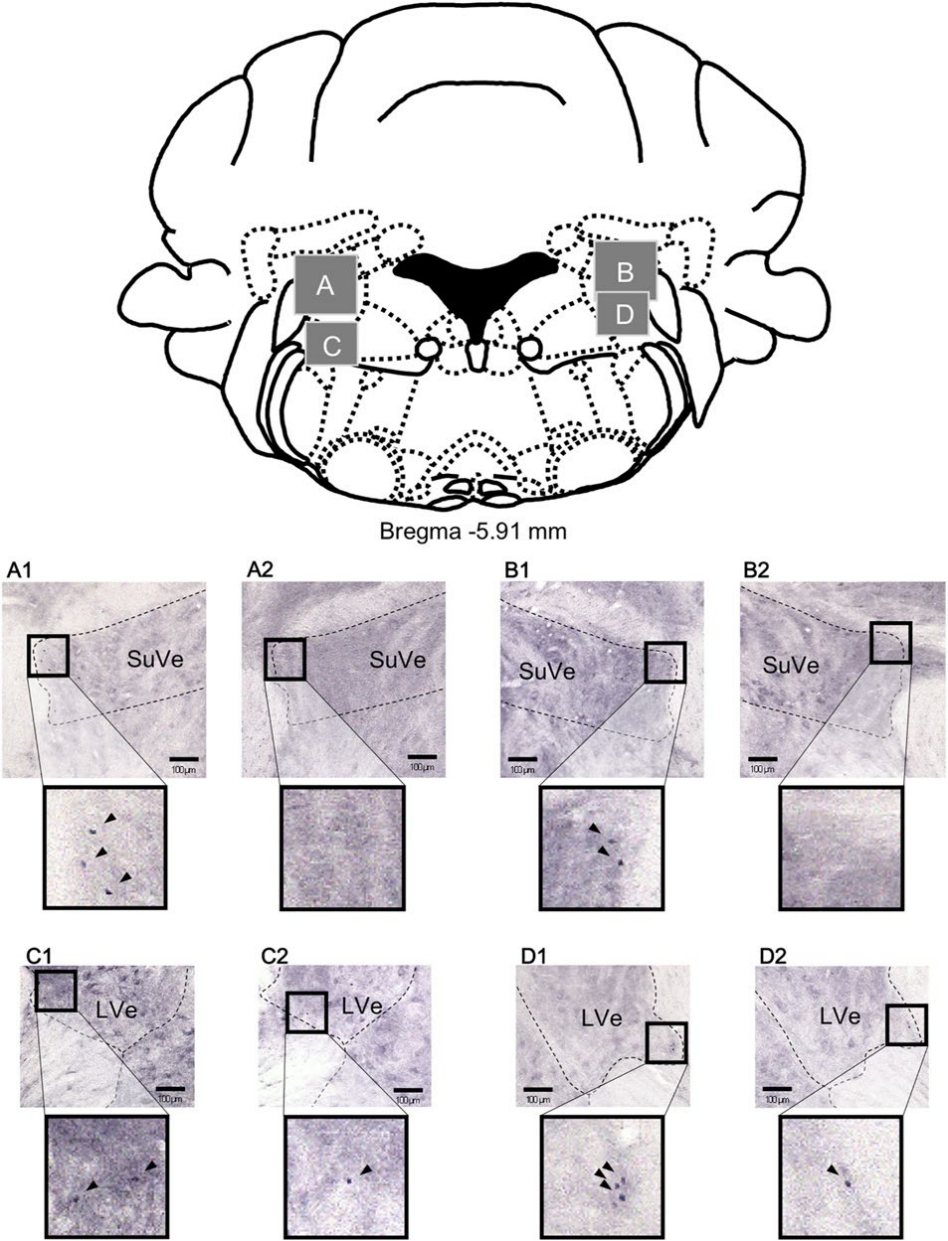
Figure 2 from [4] demonstrating increased c-Foss-positive cells in the superior vestibular nucleus as well as lateral vestibular nucleus of the medulla oblongata after 2 h exposure to low-pressure (**A1**, **B1**, **C1**, and **D1**) and under control (**A2**, **B2**, **C2**, and **D2**)

**Fig. 2:**
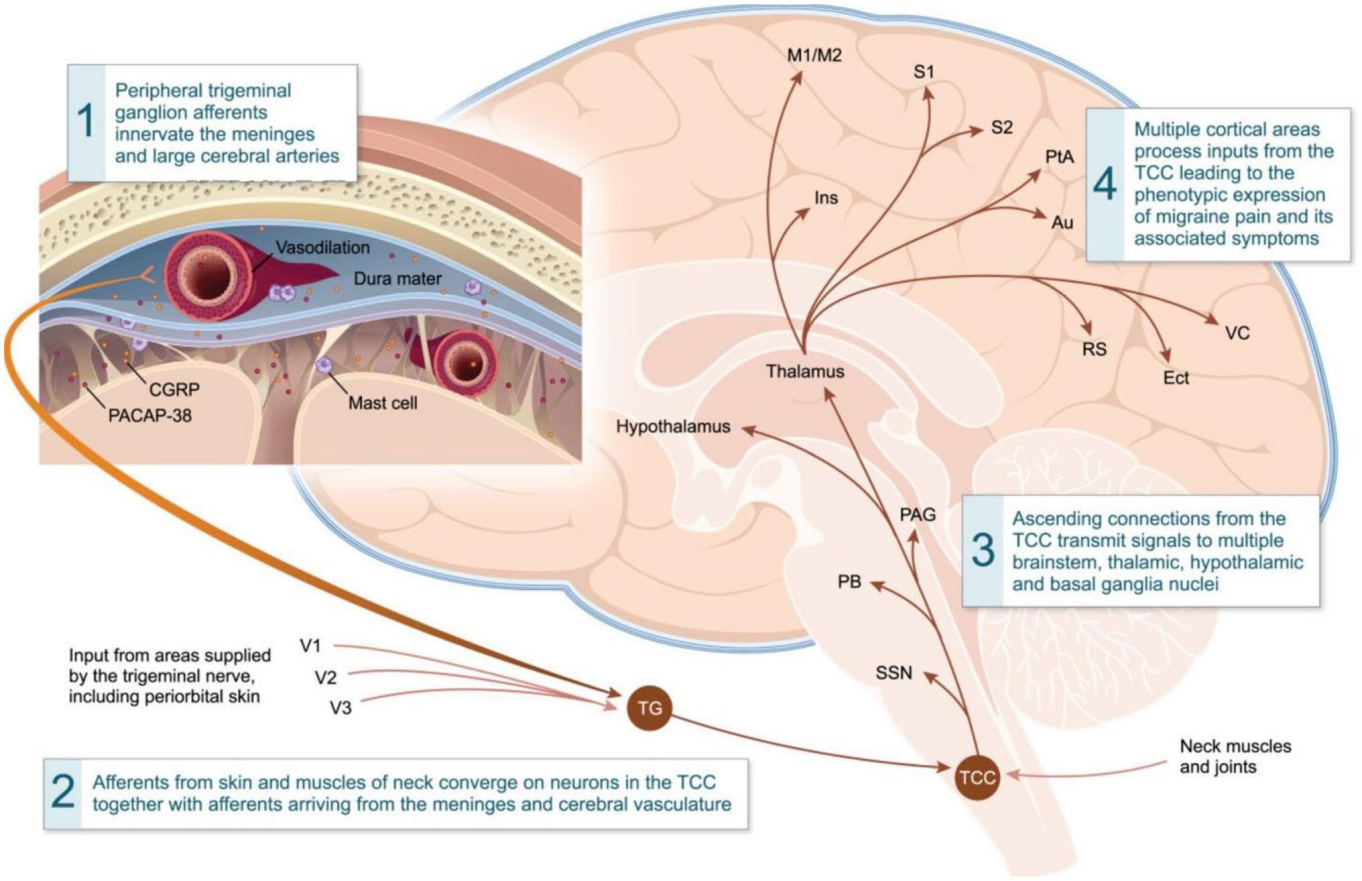
Trigeminal vascular system [6]

It is well documented in the literature that barometric pressure changes can be a trigger for patients with migraine. The exact pathophysiology behind how the barometric pressure affects headache frequency is unclear. In animal models, some studies demonstrated that reduction of barometric pressure increased pain, possibly via increasing expression of c-Foss in the superior vestibular nucleus of mice as shown in Fig. 1 [4]. Rat chronic pain models have shown that low barometric pressure can induce pain. This could be mediated through the spinal trigeminal nucleus (Fig. 2), increased sympathetic nerve activity, constriction of nerves, or a barometric pressure sensor in the inner ear [5, 6].

The association between barometric pressure changes and migraine occurrences remains controversial. In a pilot study performed by Funakubo et al., 15 healthy subjects exposed to 8-min phases of low barometric pressure in a climate chamber experienced headaches that started during the lowered barometric pressure and increased when returning to ambient atmosphere pressure [7].

In a retrospective observational cross-sectional study performed from December 2020 to November 2021 by Katsuki et al., a smartphone application was used to gather data on headache frequency and barometric pressures. An additional survey was performed to determine whether users had physician-diagnosed migraine or physician-diagnosed non-migraine headaches. Researchers utilized statistical and deep learning algorithms to analyze and predict hourly headaches based on weather conditions, specifically barometric pressure. 37.2% of users experienced physician-diagnosed migraine, 6.1% of users had physician-diagnosed non-migraine headaches, and 56.7% of users had not consulted physicians. The mean age was 34 years, and 89.2% users were female. The mean number of headache events per individual/year was 77.0. Overall, the result showed that low barometric pressure was associated with an increased number of headache occurrences [8•]. One older study suggests that individual variation may play into how barometric pressure affects migraine as it showed some patients with migraine were susceptible to low barometric pressure while others were susceptible to high barometric pressure [9]. One study on migraine triggers in Asia suggested that weather was a stronger trigger in Asian countries that had larger changes in weather [10].

## Temperature, Humidity, and Precipitation

In many observational studies, patients reported changes in patterns in migraine in association with temperature and humidity. A cross-sectional study conducted with 190 migraine patients and 140 tension-type headache patients explored the relationship between symptoms of their headaches and changes in temperature. Specifically in migraine patients, the exposure to hot or cold temperature did not have a significant relationship with clinical features of headaches. The study also searched for any association between hot or cold weather and patients’ age and BMI. In patients with tension-type headache, there was a significant relationship between hot or cold weather as a trigger and increased body mass index [11].

In a prospective study performed by Akgün et al., authors aimed to determine whether variables such as temperature and humidity affected headache quality in 50 episodic migraine and 50 episodic tension type headache patients. Patients were diagnosed as migraine per ICDH-3 and visited outpatient clinics between July 2014 and April 2015, at least literate, and over 18 years of age. During the study periods, meteorological parameters were obtained from the Republic of Turkey Ministry of Agriculture and Forestry showing weather conditions of Zonguldak. The relationship between episodic migraine attacks and weather parameters was evaluated through headache diaries kept by the patients. Specifically for migraine attacks, it was found that as mean age increased, the frequency of migraine attacks increased in cold weather. Compared to episodic tension type headache days, episodic migraine headache days had significantly higher mean wind speed. There was no significant statistical relationship found between air temperature and humidity and migraine attacks [12].

A prospective diary-based cohort study conducted from March 2016 to October 2017, in Boston Massachusetts meant to examine association between weather conditions and migraine onset, consisted of 98 patients with episodic migraine who kept diaries about duration and resolution of migraine attacks. If the migraine attack was resolved, further questions were asked to characterize the migraine attack and alleviating factors. Weather data such as ambient temperature, relative humidity, and barometric pressure was obtained from Boston Logan Airport Weather station. In this study, 49% of participants reported weather as the trigger for their migraine attacks. The 26.5% increase in relative humidity was associated with 28% higher odds of migraine attack onset. However, such association was only observed in warm seasons from April to September.

This study also found no association of temperature and barometric pressure with migraine attack onset [13].

A previously aforementioned study by Katsuki et al. also found that rainfall was associated with increased headache events [8•].

## Air Mass, Wind Speed, and Lightning

Like temperature and humidity, there is inconsistent report on how air mass and wind affect headache patterns. In a study performed across emergency departments in North Carolina over 7 years, the analysis showed that there was no statistically significant association between migraine headache emergency visits and changes in air mass types. In addition, the correlation analysis showed that as the changes in atmospheric pressure increased, the frequency of emergency visits for migraine related reasons decreased [14].

In numerous people with migraine in southern Canada, it had been reported that warm westerly winds called chinooks were a frequent cause of their migraine attacks. Cooke et al. performed individual and multiple logistic regression analysis to determine if chinook affected migraine attack onset. The calendar day during the study period was named as chinook, prechinook, or nonchinook day according to weather data provided from Environment Canada. The result showed that there was a statistically significant increase in migraine attack onset on prechinook and chinook days. Furthermore, on chinook days, relative risk of migraine attack onset was increased on high-wind chinook days, which were noted to be days with wind velocity of greater than 38 km/h. However, due to the presence of possible confounding factors, such as age, and presence of identical subjects that reported migraine attack onset on both prechinook and chinook days, the authors suggest weather effects on headache could be independent [15].

Thunderstorms have been reported to worsen migraine attack frequency among those with migraine. Martin et al. performed a cohort study in Ohio and Missouri where they collected patient headache diaries that indicated presence and absence of migraine attack, peak intensity rated from 0 to 10, and associated symptoms including nausea, vomiting, photophobia, phonophobia. The authors emphasized that thunderstorms are a combination of many weather events including changes in temperature, humidity, barometric pressure, and wind speed. Among these factors, authors focused on electromagnetic signals that indicated “cloud-to-ground lightning strikes.” Overall, the result of the study indicated that the frequency of migraine increased by 31% on lightning days versus 28% on non-lightning days. Additionally, new onset migraine attacks increased by 23% on lightning days [16].

## Other Weather Factors

There is limited literature supporting statistically significant relationship between migraine attacks and a single meteorologic factor such as air temperature, humidity, pressure, wind direction, and sunshine duration [12]. Although cloudy days have been self-reported as migraine attack triggers [17], no formal studies were found exploring clouds and migraine.

## Weather and Pain

Analogous to how migraine patients believe weather can trigger their migraine attacks, patients with other types of pain also commonly report weather as a pain trigger. Many studies have attempted to investigate the relationship between general pain and weather, but there have been mixed results. One study found that relative humidity, wind speed, and lower atmospheric pressure are significantly positively associated with pain severity even when mood and exercise were controlled for. However, the predictive value of these weather events is just 20%, hardly implicating that the weather events are directly causal of pain [18]. Yet an astonishing 62–97% of people hold that weather triggers their pain. For such a strong belief, the evidence is severely lacking. One article that analyzed various studies of weather and pain found that 63% of studies that had been conducted on the topic found a positive association between the two. However, many of these associations were not very strong and many authors concluded the results were hardly of any significance. A point must be made that the conclusions of these various studies might undermine the significance of the weathers’ effect on pain [19]. It has been well established that pain is a multifactorial entity, therefore one would not expect to find that weather is unilaterally responsible for causing pain events but rather that it could be a contributing factor [20].

One study looked at how weather factors can affect rheumatological disease pain. Two variables were considered, number of painful joints and number of swollen joints. Statistically significant results showed humidity in the winter was associated with joint tenderness and pain. Additionally, barometric pressure and temperature in the summer were both associated with joint pain. This study did have some limitations such as study participants traveling to other destinations in which the weather was unknown for some period of time [21].

Another rheumatologic condition that patients report pain in relation to weather is fibromyalgia. Similarly, there was a limited number of studies that reported weather as a symptom trigger or predictive factor. In a review article by Fagurland et al., 48 subjects (45 females and 3 males) participated in a randomized clinical trial that tested the effect of transcranial direct current stimulation. The subjects provided numeric scores (0–10) over SMS for pain and emotional measures (depression, anxiety, intensity of pain, etc.) over a 30-day period prior to treatment. In conjunction, authors obtained relevant meteorological data from Norwegian Meteorological Institute and performed statistical analysis to see the association of barometric pressure, air temperature, and relative humidity with pain related to fibromyalgia. Overall, the result showed that the impact of weather on pain scoring was significant. For instance, higher pain intensity and unpleasantness were associated with lower barometric pressure. While stress could be a confounding factor, the authors indicate that stress and low barometric pressure independently affect patients in fibromyalgia. Due to such reasons, weather could not be used as a clinical predictor for worsening disease manifestation [22].

## Weather and Serotonin and Calcitonin Gene-Related Peptide

One neurotransmitter that is heavily implicated in migraine is serotonin [23]. There are many studies that demonstrated that during a migraine attack, 5-hydroxyindoleacetic acid, the main metabolite of serotonin, increased in the urine while platelet serotonin concentration decreased. Drugs that agonize serotonin receptors can be quite successful at aborting migraine attacks [24]. Many studies looking at the relationship between weather and serotonin are dated, but their importance should not be underscored. One study from the 1970s suggested that weather can be sensed by those with migraine disease 1–2 days before it arrives through the formation of “positive ions” [25]. A 2021 study speculates that this increase in the ratio of positive air ions to negative air ions subsequently increases serotonin in the body. This largely gives support to a theory postulating that “serotonin irritation syndrome” could be responsible for triggering a migraine attack [5]. Another study also from the 1970s showed that when the weather changed from “normal” to anything else, serotonin is increased for the duration of the new weather front and returns to baseline levels when the weather is once again “normal [18].” It is also true that the human brain produces more serotonin in the summer than in the winter [19]. Given the above evidence, it seems reasonable to propose that weather fluctuations might affect migraine through neurotransmitters in congruence to how hormonal mechanisms influence the phenomenon of menstrual migraine [20].

There is limited literature on how calcitonin gene related peptide (CGRP) production is affected by weather. However, it is widely known that a drop in barometric pressure leads to decreased oxygenation in the air. In a report by Frank et al., the authors performed longitudinal measurements of plasma CGRP levels in 30 subjects with diagnosis of episodic migraine with or without aura at baseline as well as during hypoxic challenges. The experiment was conducted in a normobaric hypoxic chamber and for hypoxic challenges, FiO_2_ in the chamber was lowered by 12.6% to mimic 4500 m above sea level. The result showed that the baseline plasma CGRP was highly variable from one individual to another. Upon prolonged hypoxic challenge for about 6 h only, plasma CGRP level was increased from baseline with statistical difference [26•].

## Discussion

One must be conscious of the difficulty incurred when attempting to measure weather variables accurately to study them. Since weather variables do not occur in sole isolation of one another, it becomes difficult to, for instance, assess how wind speed can affect pain while holding temperature exactly constant. Additionally, other factors in addition to the weather condition can contribute to migraine attack occurrences.

One study assessing the relationship between weather and pain did find that mood was a confounding variable as it has its own statistically significant relationship with pain events [20].

Additionally, it should be considered that description of migraine attacks is subjective based on an individual's pain threshold. The baseline thresholds can vary widely daily and can be affected by any number of events, like a woman’s menstrual cycle. When a woman is menstruating, her threshold for pain is lower than when she is not. Therefore, it must be understood that a migraine attack will very rarely be caused by one single trigger, and the number of triggers that will be required to break the threshold of the migraine attack will be directly influenced by the individual's pain threshold at that time [27]. All in all, the effect of weather on migraine attack occurrences is complex, and there is no straightforward cause and effect pattern.

Mechanisms underlying the relationship with weather changes and migraine are convoluted and not completely understood. There are more clear examples of migraine triggers such as changes in estrogen during the menstruation cycle. Extreme weather variations such as the Chinook winds show much greater statistical significance with migraine than more common and less extreme weather variations [15]. Triggers can be difficult to attribute to a cause-and-effect relationship due to many variables, including timing of exposure to attack. They are more likely to contribute to an attack when experienced in conjunction with other triggers. It is a so-called “perfect storm” effect. Not all individuals appear to have the same triggers [28].

Is it possible that migraine patients in the prodrome phase are more sensitive to changes in weather and thus feel like the weather has triggered a migraine attack? This idea was explored in detail by Karsan et al. in a paper looking at the overlap between migraine triggers and premonitory symptoms [29]. This could help explain why those with migraine do not tend to be good predictors of their triggers. When comparing people who do and do not claim to have the ability to tell how weather is affecting their migraine patterns, there is no difference in actual predictive potential between the two [30, 31].

Limitations exist in most of the studies reported in this article. Given most are retrospective surveys and cross-sectional studies that rely on patients’ diaries, these are susceptible to reporting bias and sample size is small. Randomized controlled studies are extremely difficult to perform with weather, so a causal relationship between weather and migraine occurrences is not able to be established.

## Conclusion

Weather information can be acquired from local weather stations or weather apps and used as a guide to predict migraine attacks; however, because there is no clear causal relationship between weather conditions and migraine attacks, it is recommended that patients keep in mind that other triggers can affect migraine in addition to weather. Hence, it would be advised that those with migraine who are negatively impacted by weather conditions manage other migraine contributors such as avoiding additional controllable triggers. In addition, patients can keep their acute medications close by or even pre-emptively treat if they are having any prodromal symptoms during certain weather events that might be triggers for them. Overall, further research is needed to better elucidate the relationship between weather conditions and how they affect migraine. It would be of particular interest to see how weather conditions impact clinical management such as affecting CGRP levels in the blood and if weather disrupts certain daily routines employed by patients to help control migraine such as morning exercise, clinic appointments, or picking up medications. Further studies could investigate if acetazolamide could be used as treatment for weather related migraine attacks in a similar fashion to how it is used for altitude headache [32].

## Data Availability

Not applicable.
